# Epigenetics in a Spectrum of Myeloid Diseases and Its Exploitation for Therapy

**DOI:** 10.3390/cancers13071746

**Published:** 2021-04-06

**Authors:** Michael Maher, Jeannine Diesch, Marguerite-Marie Le Pannérer, Marcus Buschbeck

**Affiliations:** 1 Cancer and Leukaemia Epigenetics and Biology Program, Josep Carreras Leukaemia Research Institute (IJC), Campus Can Ruti, 08916 Badalona, Spain; mmaher@carrerasresearch.org (M.M.); jdiesch@carrerasresearch.org (J.D.); mlepannerer@carrerasresearch.org (M.-M.L.P.); 2 Program for Predictive and Personalized Medicine of Cancer, Germans Trias i Pujol Research Institute (PMPPC-IGTP), Campus Can Ruti, 08916 Badalona, Spain

**Keywords:** epigenetics, chromatin, epigenetic regulators, clonal hematopoiesis of indeterminate potential (CHIP), myelodysplastic syndromes (MDS), acute myeloid leukemia (AML), secondary acute myeloid leukemia (sAML)

## Abstract

**Simple Summary:**

The genome is stored in the limited space of the nucleus in a highly condensed form. The regulation of this packaging contributes to determining the accessibility of genes and is important for cell function. Genes affecting the genome’s packaging are frequently mutated in bone marrow cells that give rise to the different types of blood cells. Here, we first discuss the molecular functions of these genes and their role in blood generation under healthy conditions. Then, we describe how their mutations relate to a subset of diseases including blood cancers. Finally, we provide an overview of the current efforts of using and developing drugs targeting these and related genes.

**Abstract:**

Mutations in genes encoding chromatin regulators are early events contributing to developing asymptomatic clonal hematopoiesis of indeterminate potential and its frequent progression to myeloid diseases with increasing severity. We focus on the subset of myeloid diseases encompassing myelodysplastic syndromes and their transformation to secondary acute myeloid leukemia. We introduce the major concepts of chromatin regulation that provide the basis of epigenetic regulation. In greater detail, we discuss those chromatin regulators that are frequently mutated in myelodysplastic syndromes. We discuss their role in the epigenetic regulation of normal hematopoiesis and the consequence of their mutation. Finally, we provide an update on the drugs interfering with chromatin regulation approved or in development for myelodysplastic syndromes and acute myeloid leukemia.

## 1. Introduction

Myelodysplastic syndromes (MDS) are part of a spectrum of clonal myeloid diseases starting with the asymptomatic expansion of mutated hematopoietic stem cell (HSC) clones and frequently ending with transformation to full-blown secondary acute myeloid leukemia (sAML) [1]. The evolution and progression of MDS and sAML is intimately linked to changes in the regulation of chromatin function and epigenetics. First, effector enzymes with epigenetic regulatory functions are among the most commonly mutated genes in MDS and AML [2,3]. Second, epigenetic abnormalities co-occur with genetic and cytogenetic changes in MDS and sAML, and together, contribute to the full manifestation of the disease [4]. Indeed, the accumulation of epigenetic changes has been suggested to represent a tipping point to transformation to sAML [1]. The fact that epigenetic changes are reversible has provided the rationale for developing therapies that target epigenetic regulators.

In this review, we provide a short background on MDS and sAML as part of a spectrum of clonal myeloid diseases with increasing severity. We only touch on the clinical aspects of disease management that have been reviewed elsewhere [5]. After a brief introduction of the concept of epigenetics and its relation with chromatin modifications, we discuss those epigenetic regulators that are affected by mutations in MDS and sAML. Finally, we summarize current and emerging epigenetic drugs that are used and tested for the treatment of these myeloid diseases. Importantly, epigenetic alterations also contribute to other hematologic diseases, and we would like to refer to recent reviews discussing these aspects in lymphoma [6] and other types of AML not related to MDS [7].

## 2. CHIP-MDS-sAML—A Spectrum Myeloid Diseases

**Figure 1:**
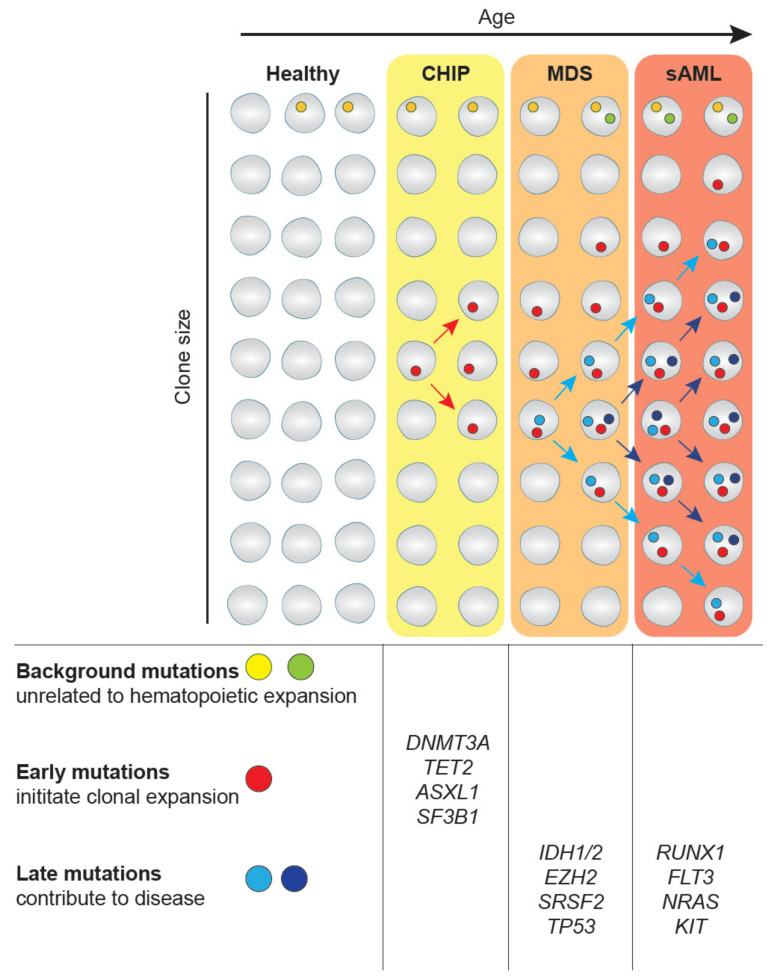
Clonal hematopoiesis in myelodysplastic syndromes (MDS) and transformation to secondary acute myeloid leukemia (sAML). Mutations in hematopoietic stem cell (HSC) clones occur at any time of our life as part of the aging process. While most mutations are background mutations that do not affect cellular properties, some mutations provide an advantage to HSCs, such as increased self-renewal. These mutations drive clonal expansion and the eventual development of the asymptomatic clonal hematopoiesis of indeterminate potential (CHIP). The further expansion frequently driven by the acquisition of additional genetic alterations can lead to MDS. The gain of additional driver mutations can further lead to transformation to sAML. This figure has been inspired by [7].

The expansion of clonal populations of blood cells from a single hematopoietic stem cell (HSC) with one or more somatic mutations is divided into two categories age-related clonal hematopoiesis (ARCH) and clonal hematopoiesis of indeterminate potential (CHIP). ARCH describes broad recurrently occurring mutational events that can cause clonal hematopoiesis and lead to age-related pathologies, including inflammation, cancer mortality, as well as hematological malignancies [6]. On the other hand, CHIP is associated with detectable somatic clonal mutations in leukemia-driver genes with a variant allele frequency (VAF) of 2% or greater [8] (Figure 1). Individuals with CHIP show normal peripheral blood counts and no evidence of WHO-defined criteria for a hematological malignancy or other clonal disorders [9]. Mutations that also occur in MDS and sAML have been observed in healthy, mainly elderly populations as part of population-based studies [10,11]. CHIP-related mutational burden appears to increase with age, as CHIP is present in 10–15% of individuals aged over 70 years [1]. Interestingly, the most frequent mutations in CHIP affect the epigenetic regulators TET2, DNMT3A and ASXL1 and the splicing factor SF3B1. Individuals with CHIP have an increased risk of developing diseases of the lymphoid and myeloid lineage, including MDS. This happens when mutations increase the fitness of HSC clones allowing them to expand among the bulk HSC population, eventually resulting in clonal dominance. If mutations are coupled with reduced differentiation capacity, the expansion of mutated HSCs can lead to reduced generation of mature blood cells in one or several lineages (Figure 1). The current challenge lies in understanding how CHIP predisposes to developing disorders. For a more thorough discussion of CHIP and its consequences, please see recent reviews [6,8].

MDS is the most frequent hematopoietic disorder in the elderly [12,13]. Advanced age is the main contributing risk factor of acute myeloid malignancies, with the median age of diagnosis at around 70 years and 92% of MDS patients aged over 50 years [14,15]. MDS is characterized by the expansion of mutant HSC clones at the expense of normal hematopoiesis leading to low blast cell counts, but a substantial reduction of numbers of mature blood cell types referred to as cytopenias. Consequential symptoms are fatigue due to anemia [16], recurring infections related to neutrophil dysfunction [17] and autoimmune abnormalities, such as rheumatic heart disease [18]. 

Around 30% of MDS patients transform to sAML [19], which is characterized by further increases in blast cell counts above 20% in the bone marrow [20]. On the genetic and molecular level, sAML mutant HSC clones have acquired additional driver mutations that convert them into full leukemia stem cells (LSCs). These genetic alterations differ to some extent from other AML subtypes [21]. De novo AML occurs without any previous neoplasm, is more common in younger patients and is associated with better overall survival [22]. Compared to CHIP and early-stage MDS, LSCs in sAML and late-stage MDS have acquired mutations that confer uncontrolled growth, such as *NRAS*, and inhibition of apoptosis, such as *TP53*. Together with epigenetic abnormalities, these oncogenic mutations cause blast cell numbers to increase and inhibit differentiation, which is characteristic of the MDS-to-sAML transformation [1]. Furthermore, an abnormal stem cell niche in the bone marrow may favor the outgrowth of mutant clones and thus contribute to the disease [23,24].

In summary, MDS and sAML are part of a spectrum of clonal diseases affecting the myeloid lineage that can arise from CHIP. Mutations in epigenetic regulators are early events and provide a yet not fully understood function in disease etiology.

## 3. Modifications of Chromatin Are the Molecular Basis of Epigenetic Regulation

A modern definition of epigenetics refers to a level of memory affecting gene function without changes in DNA sequence. In cells, DNA is stored in the nucleus and compacted into chromatin. On the macroscopic level, we can distinguish two degrees of compaction. Heterochromatin is highly compacted and is mostly transcriptionally repressed. In contrast, euchromatin is a more “open” structure that allows gene transcription to take place. Furthermore, the three-dimensional conformation of chromatin dictates the degree of interaction between promoters and regulatory elements, thus providing a layer of transcription regulation [25].

The nucleosome is the structural unit of chromatin and comprises DNA wound around an octamer complex of core histones: H2A, H2B, H3, and H4 [26]. Writer enzymes modify DNA and histones and include acetyltransferases, methyltransferases, kinases and ubiquitinases. Enzymes that remove these modifications, termed “erasers”, include deacetylases, demethylases and deubiquitinases [27]. A large majority of modifications occur on histone tails that are at an accessible position outside of the core of the nucleosome. Proteins that detect these modifications are termed “readers”. For instance, proteins with bromo- and extra-terminal domains (BET) recognize lysine acetylations on histones. The interplay between writers, readers and erasers can affect the function of the surrounding chromatin, including gene regulation and thus are part of the epigenetic machinery. Chromatin modifications mediate influence gene regulation in at least two ways. First, they can affect the relative accessibility of transcription factors to DNA [27]. Second, they can affect higher-order chromatin structures that alter the relative distance and contact frequency of regulatory elements and gene promoters [28].

To give a few examples: histone acetylation mainly occurs at the N-terminal tails of the histones H3 and H4 are regulated by histone lysine acetyltransferases (KAT) and deacetylases (HDAC). Acetylation neutralizes the positive charge of lysine residues between neutral and positive, respectively. Acetylation is generally associated with open chromatin and active gene expression by relaxing DNA and histone interactions, whereas deacetylation is associated with a more closed chromatin structure and repressed gene expression due to stabilization of the chromatin structure [29]. The BET family of proteins, such as BRD2, BRD3 and BRD4, recognize acetylated lysine residues and enhance transcription by recruiting chromatin remodelers and other factors of the transcriptional machinery [30]. In contrast to acetylation, histone methylation does not affect the charge but rather directs a wide range of reader proteins that recruit other effector proteins, such as chromatin remodeling complexes [31,32]. Histone methylation is highly site-specific and can have activating or repressive consequences [33].

On the level of DNA, DNMTs catalyze the addition of a methyl group to cytosines in the context of CpG dinucleotides. De novo methylation is carried out by DNMT3A and DNMT3B, while DNMT1 maintains methylation states during replication [34]. Active demethylation is carried out by hydroxylation of 5-methylcytosine to 5-hydroxymethylcytosine (5-hmC) by ten-eleven translocation (TET) enzymes, such as *TET2* [35]. Passive demethylation can occur in the absence of DNMT1 activity over several rounds of DNA replication [36]. While dispersed CpGs are by default methylated, many promoters contain CpG-rich sequences that are kept unmethylated. CpG methylation can interfere with the binding of some transcription factors to their respective binding motifs, thus constituting a repression level [37].

Taken together, an important part of epigenetic regulation is based on reversible modifications of DNA and histones. In the next section, we only discuss the function of those epigenetic regulators that are recurrently mutated in CHIP, MDS and AML. For more detail and further reading on the role of other epigenetics in hematopoiesis, we would like to point towards several recent reviews from colleagues [38,39,40].

## 4. Epigenetic Regulators Frequently Mutated in Myeloid Diseases and Their Function

Recurrent mutations in CHIP, MDS and sAML affecting genes involved in epigenetic regulation include regulators of DNA methylation, histone modifiers and elements regulating higher-order chromatin architecture [2,41]. For these groups of genes, we discuss their normal role in hematopoiesis and the consequences of their mutations in the disease (summarized in Table 1). Again, we focus on MDS and sAML but also discuss selected insights from other types of AML.

### 4.1. Mutations Causing Aberrant DNA Methylation—TET2, DNMT3A, IDH

Advances in genome-wide DNA methylation studies have revealed distinct DNA methylation patterns at different stages of differentiation during hematopoiesis that demarcate myeloid and lymphoid lineage decisions [86,87]. In general, myelopoiesis is associated with a reduction of methylation marks. Genes methylated at their promoters in myeloid progenitor cells of mice were reported to become unmethylated in a lineage-specific manner. Examples are the neutrophil-specific gene, *Mpo,* encoding myeloperoxidase and *Cxcr2* that encodes a chemokine to allow chemotaxis [88]. In contrast, lymphopoiesis depends on the maintenance of DNA methylation, as evidenced by a reduction in lymphoid progeny in mice with reduced Dnmt1 activity [88]. A principal characteristic of HSC is its life-long ability to self-renew. When DNMT1 activity is removed in mice, HSC and progenitors were reduced in the bone marrow, and differentiation patterns were disrupted, suggesting maintenance of DNA methylation plays a direct role in regulating HSC self-renewal and cell fate decisions [88]. Aberrant DNA methylation can often be seen in MDS and AML and is thought to drive disease progression [89]. In particular, mutations in *TET2* and *DNMT3A* are frequently observed in the early stages of CHIP [9] and highlight the important role of aberrant DNA methylation, and not just hyper- or hypomethylation, in the contribution to myeloid malignancies [90].

DNMT3A establishes de novo DNA methylation, and it is thought that heterozygous mutant *DNMT3A* acts as a dominant-negative over wild-type *DNMT3A*, thereby reducing overall methyltransferase activity [49]. HSC of conditional *Dnmt3a*-knockout mice displays reduced differentiation capacities, while their self-renewal was elevated, which resulted in an accumulation of *Dnmt3a*-null HSCs in the bone marrow [50,51]. Similarly, in xenograft models, human *DNMT3A*-mutant HSCs demonstrated an advantage compared to wild-type HSCs, highlighting their contribution to a pre-leukemic state prior to the acquisition of additional mutations [52]. Indeed, *DNMT3A* mutations are one of the first ones to arise [53,54].

TET enzymes carry out antagonistic biochemical functions to DNMT3A [78]. TETs promote demethylation in an indirect manner involving oxidation of the methylated cytosine and base excision [79]. Deleterious *TET2* mutations are common in hematologic malignancies, with 30–50% in patients with MDS and myeloproliferative neoplasia and 30% in sAML patients [80]. *TET2* deficiency causes widespread hypermethylation in mice, where upregulated oncogenes and downregulated tumor suppressor genes may have contributed to the observed leukemogenesis [81]. Deletion of *TET2* in CD34+CD38+ hematopoietic progenitor cells resulted in increased monocyte expansion, suggesting a role in myeloid differentiation or lineage commitment [82]. In various studies, the mutational status of *TET2* has been associated with poor prognosis [83,84], while others could not demonstrate this association [80,85].

Isocitrate dehydrogenase (IDH) is a key enzyme in the citric acid cycle that catalyzes the conversion of isocitrate to 2-ketoglutarate, which is an important cofactor for TET enzymes and some histone demethylases [60]. *IDH* mutations are neomorphic mutations that change the enzymatic capacity resulting in the production of elevated levels of 2-hydroxyglutarate (2-HG), which acts as a competitive inhibitor of TETs and other 2-ketoglutarate-dependent enzymes, leading to a widespread increase in histone and DNA methylation [61,62]. IDH mutations block differentiation and promote LSCs to proliferate [63]. Mutations in *IDH1* and *IDH2* have been identified in around 5% of MDS cases [64], 9.7% of sAML and 20% of AML patients [60]. IDH1 mutations are less common than IDH2 mutations [64]. In *IDH1*, mutations can often be found on arginine R132 in the form of a cysteine (R132C) or histidine (R132H) substitution. In *IDH2*, the mutations affect arginine R140 or R172 replaced by glutamine (R140Q) or lysine (R172K), respectively. In myeloproliferative neoplasms and high-risk MDS, *IDH* mutations were linked to disease progression [65]. In contrast, in AML, the prognostic impact of *IDH* mutations could not be clearly determined and may depend on the specific point mutation and the presence or absence of co-mutations [60].

### 4.2. Dysregulation of Histone Modifications—EZH2, RUNX1, BCOR, ASXL1

The multimeric polycomb repressive complexes (PRC) 1 and 2 are histone writers that contribute to transcriptional silencing. PRC2 is responsible for all di- and tri-methylation of lysine 27 of H3 (H3K27me2/me3) that is mediated by its subunit EZH2 [91,92]. During lymphopoiesis, high expression levels of *EHZ2* are associated with proliferating cells suggesting a role in lineage-specific cell cycle regulation [55]. H3K27me3 mediates the recruitment of PRC1 that mono-ubiquitylates H2A at lysine 119, inhibits transcriptional elongation and promotes chromatin compaction [38]. Interestingly, the PRC2-induced H3K27me3 mark is offset by the trithorax group (trxG), which mediates the activating H3K4me3 mark associated with open chromatin and gene activation [93]. Genes in loci that contain both marks are so-called “bivalent” domains that indicate flexible activation and repressive mechanisms. HSC contains many such bivalent genes [94]. Genome-wide changes of gene expression and histone modifications have shown HSC genes are “primed”’ for subsequent activation or repression during lineage commitment [95]. In this way, PRCs are thought to contribute to HSC self-renewal and maintenance of pluripotency by dynamically repressing cell fate regulators during hematopoiesis [56]. Mutations in *EZH2*, *BCOR*, *ASXL1* and *RUNX1* affect the function of PRCs.

Both loss and gain-of-function mutations of *EZH2* are found in hematological disorders indicating a context-dependent function of EZH2 as an oncogene or tumor suppressor [56]. In MDS, primarily inactivating mutations of *EZH2* occur in around 5% of patients [2] and are associated with poor prognosis [57] but not with progression to AML [96]. In de novo AML, loss-of-function mutations of *EZH2* are less frequent and occur in 1–2% of patients [21]. Mechanistically, loss of *Ezh2* in mice has been shown to promote MDS development by activating inflammatory cytokine responses resulting in impaired HSCs differentiation [58]. On the other hand, *Ezh2*-deficient mouse models have demonstrated the requirement of EZH2 for developing myeloid malignancies, including MLL-AF9 AML, in which Ezh2 mutation or deletion causes a loss of LSCs and an increase in differentiation [59].

While EZH2 is a component of PRC2, BCOR is a component of a variant of the PRC1 complex [46,97]. *BCOR* loss-of-function mutations occur in about 5% of cases of MDS and 9% of sAML patients and are associated with a poor prognosis [2,47]. Bcor loss results in myeloid progenitor expansion and the presence of oncogenic *Kras^G12D^* promotes leukemogenesis in mice [48].

*ASXL1* forms a complex with BRCA1-associated protein 1 (BAP1) that physically interacts with PRC2 and deubiquitinylates histone H2A [42]. *ASXL1* mutations lead to reduced levels of *ASXL1* and are associated with a global reduction of PRC2 recruitment and H3K27me3 [42]. *ASXL1* is mutated in approximately 20% of MDS patients, thus representing one of the top mutated genes [2]. In AML, *ASXL1* mutations occur in 6–30% of patients and correlate with advancing age [43,44,45].

Mutations in *ASXL1, EZH2* and *BCOR1* are associated with mutations in the gene encoding the transcription factor *RUNX1* [2]. With more than 50 reported translocations and various point mutations, *RUNX1* is one of the most frequently mutated genes in AML [66,67]. In MDS, *RUNX1* mutations occur in 10–20% of patients [68]. HSC self-renewal is disrupted in animals with mutated *RUNX1* [69]. *RUNX1* regulates the *PU.1* gene, which is involved in developing all hematopoietic lineages. Disruption of normal *RUNX1* activity results in *PU.1* downregulation with various lineage-specific consequences, including an increased percentage of granulocytes in the bone marrow of mice [70]. While it is not fully clear how *RUNX1* mutations synergize with mutations related to PRC function in disease, it is interesting to point out that RUNX1 protein can physically interact with PRCs and promote gene repression through their recruitment to gene promoters [71].

### 4.3. Altering Chromatin Structure—The Cohesin Complex

Somatic mutations affecting the cohesin complex have been identified in several diseases, including MDS and AML [98]. The cohesin complex consists of the core subunits SMC1, SMC3 and RAD21, which associate with either STAG1 or STAG2. One of its important functions is to align and stabilize sister chromatids during metaphase crucial for DNA replication, DNA repair and mitosis [72]. In addition, cohesin has an important role in the regulation of genome folding in interphase cells [73]. Loss-of-function cohesin mutations, mainly in the *STAG2* gene, were detected in 10–15% of MDS and 20% of sAML patients and are associated with poor survival [74]. Interestingly, in several human leukemic cell lines, low expression of cohesin was observed, although no mutation could be identified [74]. On the mechanistic level, reduced cohesin function leads to changes in gene expression, possibly as a direct consequence of changes in chromatin architecture [75]. In particular, reduced sensitivity to inflammatory signals may affect the function of HSCs [76].

In conclusion, with mutations affecting cohesin, histone-modifying PRCs and the DNA methylation machinery, several central epigenetic mechanisms are perturbed in MDS and sAML. The common denominator of these mutations in disease is that they disrupt normal hematopoietic differentiation and promote the expansion of altered HSCs [74], thereby contributing to disease progression. The challenge for the field now is to identify specific vulnerabilities of mutant cells that can be exploited for therapeutic strategies aiming at synthetic lethality. An exciting example is a recent demonstration that cohesin mutant cells are hypersensitive to inhibitors of the DNA repair pathway [77].

## 5. Epigenetic Drugs

**Figure 2:**
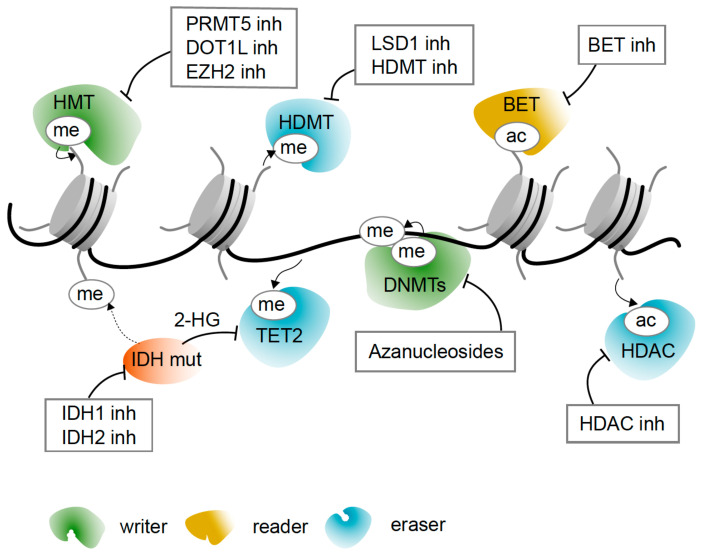
Overview of epigenetic drugs for MDS and sAML therapy. Many epigenetic enzymes are involved in the regulation of gene function. These can be broadly classified into “writers”, which add specific marks to core histones, namely methyl (me) and acetyl (ac) groups; “readers”, which identify the marks; and “erasers”, which remove these marks. DNA methyltransferases (DNMTs) methylate DNA, thereby silencing certain tumor suppressor gene expression. Hypomethylating agents, such as azanucleosides, are thought to reduce DNMT activity, thus reactivating silenced genes. Histone methyltransferase (HMT) inhibitors (inh) that target mutated PRMT5, DOT1L and EZH2 seek to re-stabilize perturbed histone methylation states. Histone deacetylase (HDAC) inhibitors restore histone acetylation, thus activating gene expression to promote differentiation and apoptosis. Ten-eleven translocation (TET) enzymes catalyze the demethylation of 5-methylcytosine to 5-hydroxymethylcytosine to induce active DNA demethylation. Isocitrate dehydrogenase (IDH) inhibitors reduce total serum levels of the oncometabolite, 2-hydroxyglutarate, restoring normal TET2 activity and DNA and histone methylation levels. Bromodomain and extra-terminal domain (BET) inhibitors mainly target BRD4, which normally promotes transcription of oncogenes, such as MYC, by binding acetylated histones.

Epigenetic changes are inherently reversible, making them potentially suitable for therapeutic intervention. DNA hypomethylating azanucleosides are the primary pharmacologic therapy for a subset of high-risk MDS patients that have a 50% response rate [99]. However, resistance is common and durable remission is rare. Here, we give an update on the use of azanucleosides and discuss ongoing efforts to target other epigenetic mechanisms as therapeutic strategies (summarized in Table 2 and Figure 2), as well as outline clinical trials using epigenetic drugs (Table 3).

### 5.1. Azanucleosides Are DNA Hypomethylating Agents and More

The azanucleosides, 5-azacitidine (azacitidine), and 5-aza-2′-deoxycytidine (decitabine) are nucleoside analogs that have become the mainstay of MDS treatment for intermediate- to high-risk MDS patients, who are ineligible for allogeneic hematopoietic stem cell transplantation (HSCT). It is thought that the main mechanism of action of hypomethylating agents is the inhibition of DNA methyl-transferases (DNMTs), resulting in the reactivation of gene transcription by demethylating promoter regions of tumor suppressor genes [178]. In this way, tumor suppressor gene expression is increased, along with genes related to cell differentiation and apoptosis, thereby dampening MDS progression [100]. Additionally, reactivation of endogenous retroviruses mimics a viral infection, thereby activating the innate immune response [179]. Upon azanucleoside incorporation into DNA, adducts are formed between DNA and DNMTs, which prevents DNA methylation and activates DNA damage response causing cytotoxicity [180]. Interestingly, despite its known hypomethylating effects and in contrast to decitabine, which is only incorporated into DNA, 80–90% of azacitidine is incorporated in RNA. The incorporation of azacitidine into RNA has been shown to inhibit tRNA methyltransferases leading to impaired tRNA methylation and processing [181,182]. Additionally, rRNA processing is reduced, ultimately causing a general inhibition of mRNA and protein synthesis [183]. A recent report from Cheng et al. [184] showed that RNA-dependent effects of azacitidine determine cellular sensitivity to the treatment. In azacitidine-sensitive cell lines, RNA-polymerase II interacts mainly with RNA 5-methylcytosine methyltransferases NSUN3 and DNMT2, and interaction, which is rapidly disrupted by azacitidine. In contrast, in azacitidine-resistant cell lines and specimens from azacitidine-resistant MDS/AML patients, RNA-polymerase II interacts with NSUN1, and interaction, which does not get disrupted by azacitidine.

As a derivative of the chemotherapeutic agent cytarabine, azanucleosides were originally investigated at high doses for MDS treatment until later studies reported an increased efficacy at low doses with higher remission rates and lower blast counts [100]. Subsequently, both drugs were shown to induce differentiation and act as hypomethylating agents at low doses [101,102].

The clinical efficacies of azacitidine and decitabine were later confirmed in clinical trials, which eventually led to their approval by the US Food and Drug Administration (FDA) and the European Medicines Agency approval as a treatment for MDS and AML [103,104]. Further clinical trials have shown the efficacy of azacitidine across different MDS patient groups [105,106]. Azacitidine represents the best treatment for high-risk MDS patients ineligible for HSCT [107].

Approximately only half of MDS patients respond with hematologic improvement to azacitidine treatment [105]. The response to treatment is normally apparent after less than six months of treatment [108] and seldom persists; a large proportion of initial responders eventually relapse within a two-year period [109]. The mechanisms underlying primary and secondary resistance remain open questions. Furthermore, there is a discrepancy between studies on whether azanucleoside treatment improves the overall survival of MDS patients [110,111] and whether there is a difference in the clinical efficacy between azacitidine and decitabine [185,186].

In AML, azacitidine and decitabine have also been shown to be promising. Especially in older AML patients and certain high-risk subsets, treatment with HMAs led to an advantage compared to conventional care [112]. This was also demonstrated in other studies [113,114,115,116]. While azacitidine is also FDA-approved for the treatment of AML, decitabine has only received approval by the European Medicines Agency (EMA) due to the lack of statistical significance in phase III clinical trial [113].

DNA methylation plays an important role in both regulating normal hematopoiesis and disease progression in MDS [78] and has, therefore, provided a rationale for use as a therapy marker [187]. Crucially, there has been a lack of correlation between global hypomethylation following azacitidine treatment and response. It was found that there was no difference in global methylation levels following azacitidine treatment between MDS patients resistant to azacitidine and those who achieved complete remission [117]. Initially, MDS patients with *TET2* mutations were reported to show a greater response to DNMT inhibition compared to *TET2* wild-type [118], suggesting a dependency on aberrant methylation. However, this finding has been seldom repeated, and similarly, univariate analyses examining *TET2, DNMT3A*, *IDH1*/*2*, *AXSL1*, and other risk factors, have shown no single biomarker is a predictor of response [119,188].

The limited efficacy of hypomethylating agent monotherapies and the strict inclusion criteria for HSCT highlights the need for novel drugs. Indeed, several next-generation hypomethylating agents, as well as combinatorial strategies, are currently being evaluated. These include oral azacitidine CC-486, which has been FDA-approved as maintenance therapy in AML in September 2020 [120] (https://www.fda.gov/drugs/resources-information-approved-drugs/fda-approves-onureg-azacitidine-tablets-acute-myeloid-leukemia, accessed on 6 April 2021). Additionally, Guadecitabine (SGI-110) shows promising results in phase 2 clinical trials [121]. Guadecitabine is a dinucleotide of decitabine and deoxyguanosine linked by a phosphodiester bond, which is gradually cleaved, leading to a slow-release and thus prolonged cellular exposure of its active metabolite decitabine [122,123]. The most promising combinatorial strategies include a combination with the selective BCL-2 inhibitor, venetoclax [124] and the mutant p53 reactivator, APR-246 [125,126]. The combination with venetoclax and azacitidine has been FDA-approved for the treatment of AML in adults of 75 years or older (21 November 2018, https://www.fda.gov/drugs/fda-approves-venetoclax-combination-aml-adults, accessed on 6 April 2021), while the combination with APR-246 has received the breakthrough designation in MDS patients with TP53 mutation (1 April 2020, https://www.ashclinicalnews.org/news/latest-and-greatest/fda-grants-breakthrough-designation-apr-246-mds/, accessed on 6 April 2021). Clinical trials with APR-246 in combination with azacitidine are ongoing and have shown high response rates in high-risk MDS patients, albeit only in those with the deactivating p53 mutation [126]. Venetoclax in combination with azacitidine increased response and prolonged survival compared to azacitidine treatment alone in MDS patients [124]. Overall, hypomethylating agents still represent the best treatment strategy for many high-risk MDS patients [127], and combinatorial treatment schemes hold the promise to improve response and to reduce the onset of primary and secondary resistances.

### 5.2. Targeting Histone Acetylation and Active Transcription

BET inhibitors (BETi) have gained increasing attention in recent years as potent modulators of genes involved in disease progression across several cancers [189]. BRD4 is the most studied BET protein in the context of therapeutic targets. Upon binding of acetylated histones, BRD4 promotes transcription through the recruitment of the mediator complex and in a manner involving synergy with other transcription factors and increased enhancer-promoter contacts [190,191]. Oncogenic fusion proteins involving rearrangements of mixed lineage leukemia (MLL) gene are drivers of hematological malignancies that act in a similar transcription-promoting manner [192]. MLL rearranged sub-types of leukemia have been shown to be particularly sensitive to BETi [128,129].

BRD4 was the first “druggable”’ BET protein. The BRD4-specific small-molecule BETi, JQ1 and I-BET, were first reported by independent groups in 2010 [130,131]. Inhibition is mediated by competitive binding with acetylated proteins that cause displacement of BRD4 from chromatin. BRD4 inhibition with the first generation in inhibitor JQ-1 decreased *MYC* activity in hematopoietic cell lines and caused anti-leukemic effects in mouse models of AML [132]. Similar results were obtained with the BETi I-BET151 [128]. These discoveries solidified BRD4 as a candidate target for hematological malignancies. Indeed, JQ1 has recently been shown to induce HSC expansion and recovery of the hematopoietic system in mice following stem cell transplantation [133]. It is thought that the susceptibility of AML to BRD4 inhibition may lie in the targeting of cell lineage-specific transcription factors that determine cell fate [134]. Similar results could be obtained using other BETi. For example, I-BET151 targeting BRD2, BRD3 along BRD4 showed particular sensitivity in NPM1-mutant AML in which it decreases proliferation and increases apoptosis [135].

In general, while the efficacy of BETi in hematological diseases is not completely understood, it is thought to be a result of a reduction in Myc transcription and inhibition of transcriptional elongation. BETi currently examined in clinical trials in AML and MDS patients include birabresib (OTX015/MK-8628), CPI0610 and ABBV-744 [136]. Results from phase 1 and 2 clinical trials indicate modest efficacy as a monotherapy in AML [137,138,139]. Some adverse effects, however, limit the clinical application of BETi as monotherapies, and it has been suggested that BETi could have an increased clinical benefit if included in combinatorial therapies allowing lower doses [140].

The more recently developed inhibitors targeting the histone acetylases CBP and p300 follow a similar rationale as BETi. CBP and its paralogue p300 are transcriptional co-activators as well as function as lysine acetyltransferases acetylating histones and non-histone proteins [193]. The most promising CBP/p300 inhibitor is CCS1477 from CellCentric [194,195], which is currently in phase II clinical trials for AML, MDS, prostate cancer and solid cancers.

### 5.3. Targeting Histone Deacetylation and Gene Repression

The targeting of histone deacetylases (HDACs) is an inverse strategy compared to BETi. Increased activity and recruitment of HDACs to promoters of genes involved in differentiation processes contribute to their silencing and thus promotes leukemogenesis [196]. HDAC inhibitors, such as vorinostat and panobinostat, restore histone acetylation, thus activating gene expression that promotes differentiation and apoptosis [141].

HDACs are encoded by 18 genes and divided into four classes [197]. Clinical trials for the treatment of hematological malignancies are ongoing with inhibitors of all classes of HDACs [142]. A major drawback of the first generation of HDAC inhibitors was apparent toxicity, which is considered to be brought about by simultaneous inhibition of multiple HDAC proteins causing excessive deacetylation of non-histone proteins [143]. As a consequence, more recent iterations of HDAC inhibitor development have concentrated on isoform specificity.

Depending on the stage of disease progression, HDACs have opposing functions making their targeting challenging. HDAC1 and HDAC2 behave as tumor suppressors during the initiation of acute promyelocytic leukemia, while they act as oncogenes in established leukemia cells [144]. HDAC inhibitors are approved for lymphoma and multiple myeloma, and clinical trials, both as monotherapy and in combination, are ongoing for MDS and AML [145]. Early combinatorial trials with azacitidine show promising clinical activity and safety for MDS [146]. A deeper understanding of the mechanistic consequences of HDAC inhibition is required to devise therapies that target this aspect of epigenetic regulation better.

### 5.4. Reversing Metabolic Change with IDH Inhibitors

Neomorphic mutations in the metabolic enzymes IDH1 and IDH2 are common in AML patients. These mutations indirectly interfere with epigenetic processes by producing the 2-HG metabolite that inhibits α-ketoglutarate-dependent enzymes, which include several histone demethylases and the DNA-demethylating TET enzymes. The development of compounds specifically inhibiting mutant IDH proteins has been a successful example for accelerated drug development. The small-molecule inhibitor of mutant IDH1, Ivosidenib (AG-120), has been FDA-approved for the treatment of adult relapsed or refractory AML with IDH1 mutations on 20 July 2018 [198]. It has been shown to reduce the total serum 2-HG level and induce AML cell differentiation [147]. Combinations with standard therapy or azacitidine for the treatment of primary and secondary AML are currently assessed in clinical trials. Furthermore, there are clinical trials combining ivosidenib with venetoclax with or without azacitidine. Besides ivosidenib, several other IDH1 inhibitors are currently evaluated in clinical trials, including BAY1436032 [148], FT-2102 [149], IDH305 [150] and LY3410738 [151].

In 2013, AGI-6780 was developed as the first small selective inhibitor of mutant IDH2 and showed promising results in cell lines as well as primary AML cells [152,153]. It reduces the 2-HG level and reverses the abnormal methylation of histones. Shortly after, enasidenib (AG-221) was developed, which is a selective inhibitor of IDH2 R172K and IDH2 R140Q [154,155]. Enasidenib was FDA-approved in 2017 for the treatment of adult relapsed or refractory AML with IDH2 mutations, in which it shows efficacy, and importantly, no cytotoxicity. It reduces the 2-HG levels by more than 90%, induces differentiation of AML cells both in vitro and in murine xenograft models [156,157]. Enasidenib is currently evaluated in clinical trials in combination with azacitidine as well as with standard induction chemotherapy in AML patients with IDH2 mutations.

### 5.5. Targeting Histone Methylation with Inhibitors of Histone Methylases and Demethylases

Similar to histone acetylation, histone methylation is also perturbed in hematological diseases, and inhibitors of both methylases, as well as demethylases, are being assessed. Focusing on myeloid diseases, currently studied drug targets include the protein methylase DOT1L, the catalytic PRC subunit EZH2, the protein arginine methylase PRMT5 and the lysine demethylase LSD1.

DOT1L is a histone lysine methyltransferase specific for H3K79 [199]. H3K79 methylation is associated with active transcription as well as implicated in DNA repair by recruiting Rad9/53BP1 and cell cycle regulation [158,159]. DOT1L is an important player in the development and maintenance of MLL-rearranged AML, in which abnormal recruitment of DOT1L, particularly at MLL-fusion target genes, results in H3K79 hypermethylation and thus aberrant gene expression contributing to leukemic transformation [160,161,162]. Indeed, inhibition of DOT1L using the inhibitor pinometostat (EPZ-5676) selectively kills MLL-translocated AML cells [163]. Pinometostat has recently been examined in phase I clinical trial and has shown modest clinical effects in patients with MLL translocations [164]. Currently, it is being assessed in phase II trials in combination with standard chemotherapy in AML patients with MLL-rearrangement. In addition to pinometostat, two new DOT1L inhibitors have recently been examined in vitro and in PDX mouse models of primary MLL-rearranged AML and demonstrated comparable responses as pinometostat, but a much-improved bioavailability after oral administration [165].

EZH2 inhibitors are mainly studied in lymphomas and solid tumors in which gain-of-function mutations of EZH2 occur. The most promising inhibitor is tazemetostat, an S-adenosyl methionine-competitive inhibitor, FDA approved since 2020 for the treatment of epithelioid sarcoma [200,201]. In leukemias, in which mainly EZH2 loss-of-function occurs, it has recently been shown that EZH2 loss or reduced expression can cause the acquired drug resistance to TKI and cytotoxic drugs in AML as a result of HOX genes de-repression [202]. This leads to the speculation that restoration of *EZH2* expression might allow overcoming TKI resistance. On the other hand, it highlights that the patient group treated with EZH2 inhibitors needs to be cautiously selected.

PRMT5 mediates methylation of histones at arginine residues [203]. PRMT5 is part of complexes repressing transcription, such as MBD2-NURD and N-CoR-SMRT [203]. The methylation of arginine three on histone H4 recruits DNMT3A promotes DNA methylation and thus further enhances gene silencing [204]. PRMT5 can also methylate non-histone proteins, including p53, growth factor receptors and spliceosomal proteins, such as SRSF1 [166]. Dysregulation of protein arginine methyltransferases has been found in several cancer types, and their overexpression has been linked to poor prognosis [167]. In particular, PRMT5 is overexpressed in leukemia, lymphoma and several solid cancers and is considered to promote oncogenesis [166,167]. In AML, PRMT5 has a proleukemic role through increased expression of FLT3 [168]. This is mediated by silencing of miR-29b leading to the upregulation of its target, the transcription factor Sp1, which in turn activates FLT3 expression [168]. Furthermore, PRMT5 is required for the survival of MLL-rearranged AML cells by activating the splicing factor SRSF1 [169]. Three PRMT5 inhibitors are currently in clinical trials for several solid and blood cancers. The second-generation compound GSK3326595 is currently in phase I trials for MDS and AML, but also solid tumors and non-Hodgkin’s lymphoma. In addition to monotherapy, these trials examine the combination of immune checkpoint inhibitors and azacitidine. Two PRMT5 inhibitors have entered phase I trials in B-cell non-Hodgkin lymphoma and solid tumors, but not yet in myeloid diseases [170].

LSD1/KDM1A is a histone demethylase of mono- and dimethylated lysines on histone H3, DNMT1 and p53 [205,206]. LSD1 expression is increased in various cancer types [171] and promotes proliferation while limiting differentiation [172]. In AML, including MLL-rearranged leukemia, inhibition of LSD1 abrogates the clonogenic potential, directly promotes differentiation or sensitizes to differentiation-therapy by all-trans-retinoic acid [173,174]. Several irreversible LSD1 inhibitors are currently evaluated in clinical trials, including tranylcypromine, GSK2879552, IMG-7289, INCB059872, SP-2577 and CC-90011 [175]. Especially iadademstat has shown promising results in clinical trials in patients with MLL translocation [176,177]. In addition, trials examining the combination with ATRA as well as azacitidine are currently undergoing.

## 6. Conclusions

CHIP, MDS and sAML are intimately linked to epigenetics through mutations affecting chromatin regulators and alterations in chromatin structure and its modifications. The field has now started to exploit its knowledge about epigenetics to improve the management of myeloid diseases. Epigenetic marks have great potential to serve as response predicting markers and might help to pave the way to make well-informed personalized treatment choices [207]. In particular, DNA methylation has proven to be sufficiently robust for its analysis to be implemented in the clinical diagnostic routine. The targeting of epigenetic mechanisms is a promising approach for the urgently needed development and improvement of therapies. Without treatment intervention, high-risk MDS and sAML patients have a life expectancy of about nine months [208]. Allogeneic hematopoietic stem cell transplantation (HSCT) is the only curative treatment for MDS but associated with significant morbidity [209]. A substantial number of compounds inhibiting chromatin regulators are currently being tested in clinical trials, and many more potential drug targets from the chromatin regulatory space are being evaluated in the preclinical setting. Therapeutic strategies include the direct targeting of mutated proteins, such as in the case of IDH inhibitors, or downstream dependencies as in the case of MLL-rearranged leukemias that are hypersensitive to BETi. Screens are underway that aim at identifying targets for synthetic lethal approaches with specific disease mutations. In addition, broad-spectrum epigenetic drugs without a mutated target show promising results in particular when combined with orthogonal approaches, such as chemotherapy, immune modulation, and differentiation-induction therapy. The approval of azanucleosides for the treatment of MDS and sAML was the first epigenetic therapy to reach the clinic. Combinations azanucleosides with other drugs, such as venetoclax have now started to improve clinical benefit by increasing rate, duration and extent of response.

We expect the number of clinical applications for epigenetic drugs and biomarkers to rise and that progress in the management of myeloid diseases will lead to the treatment of other liquid and solid cancers.

## Figures and Tables

**Table 1:** Mutations in epigenetic regulators in MDS and AML.

Gene	Mutation Effect on Gene	Mutational Frequency	Characteristics
ASXL1 [42,43,44,45]	Loss-of-function mutation	20% in MDS	Mutations enriched in elderly AML and sAML patients
		6–30% in AML	
BCOR [46,47,48]	Loss-of-function mutation	5% in MDS	Associated with poor prognosis
		9% in AML	
DNMT3A [49,50,51,52,53,54]	Loss-of-function mutation	13% in MDS	Thought to be initiating mutation during the pre-leukemic state
		20% in AML	Important for the balance of differentiation and self-renewal
EZH2 [55,56,57,58,59]	Loss-of-function mutation as well as gain of function mutations	5% in MDS	Thought to regulate the balance between self-renewal and differentiation
		1–2% de novo AML	In MDS associated with poor prognosis
IDH1/2 [60,61,62,63,64,65]	Gain of function	5% in MDS	Leads to the production of oncometabolite, which interferes with TET2 activity and histone demethylases
		20% in AML	IDH2 mutations are more common
RUNX1 [66,67,68,69,70,71]	Translocations	10–20% in MDS	Significantly associated with EZH2 mutations
	Loss-of-function mutation	2–20% in AML	
Cohesin [72,73,74,75,76,77]	Loss-of-function mutation	10–15% in MDS,	Mutually exclusive
		10% in AML	often associated with mutations in NPM1, TET2, ASXL1 and EZH2
TET2 [78,79,80,81,82,83,84,85]	Loss-of-function mutation	30–50% in MDS	Important for myeloid differentiation and lineage commitment
		30% in sAML	Associated with poor prognosis in some studies

**Table 2:** Current epigenetic drugs in the treatment of myeloid malignancies.

Targets/Agents	Characteristics/Mechanisms of Action
Azanucleosides [100,101,102,103,104,105,106,107,108,109,110,111,112,113,114,115,116,117,118,119,120,121,122,123,124,125,126,127]	Promote differentiation, activate the innate immune response and lead to DNA damage response causing cytotoxicity.
	Through incorporation into RNA, AZA also reduces protein synthesis and impairs DNA synthesis and repair.
	Azacitidine and decitabine are FDA-approved for the treatment of MDS.
	Oral azacitidine CC-486 FDA approved as maintenance therapy in AML.
BET [128,129,130,131,132,133,134,135,136,137,138,139,140]	Mainly BRD4 inhibitors.
	Reduce expression of oncogenes, including MYC and BCL2, thus lead to reduced proliferation and increased apoptosis.
	In clinical trials, modest efficacy and adverse effects suggesting their use in combinatorial therapy.
HDAC [141,142,143,144,145,146]	Inhibitors restore histone acetylation, promoting differentiation and apoptosis.
	Often have dual roles making their use as monotherapies difficult.
IDH1/IDH2 [147,148,149,150,151,152,153,154,155,156,157]	IDH inhibitors reduce the total serum 2-HG level and induce AML cell differentiation.
	IDH1 inhibitor Ivosidenib and IDH2 inhibitor enasidenib are FDA approved for the treatment of adult relapsed or refractory AML with IDH1 or IDH2 mutations, respectively.
EZH2 [158,159,160]	S-adenosyl methionine-competitive EZH2 inhibitor tazemetostat is FDA approved for the treatment of epithelioid sarcoma.
DOT1L [160,161,162,163,164,165]	DOT1L inhibitor pinometostat selectively kills MLL-rearranged AML cells and is in phase I clinical trial in patients with MLL translocation.
	Pinometostat has limited pharmacokinetics (requires continuous intravenous administration); thus, new DOT1L inhibitors are currently being assessed in vitro and in PDX models.
PRMT5 [166,167,168,169,170]	PRMT5 inhibition has anti-leukemic effects in AML due to the downregulation of *FLT3* expression.
	PRMT5 inhibition induces alternative splicing and downregulation of proteins required for proliferation.
LSD1 [171,172,173,174,175,176,177]	LSD1 inhibition abrogates the clonogenic potential and induces differentiation of MLL-rearranged AML as well as sensitizes AML cells to differentiation induced by all-trans-retinoic acid.

**Table 3:** Ongoing or recently completed clinical trials using epigenetic drugs in the treatment of myeloid malignancies. Allo-HSCT, allogeneic hematopoietic stem cell transplantation; AML, acute myeloid leukemia; ATRA, all-trans retinoic acid; CMML, chronic myelomonocytic leukemia; DLBCL, diffuse large B-cell lymphoma; MDS, myeloid dysplastic syndrome; MPN, myeloproliferative neoplasms; R/R, relapsed or refractory; sAML, secondary acute myeloid leukemia.

Targets/Agents	Drug Name	Diseases	NCT Number	Phase Trial	Combination	Completion Date
HMAs	Azacitidine	IDH1-mutant AML and MDS	NCT03471260	Phase I/II	Venetoclax and ivosidenib	2021
		TP53-mutant AML and MDS	NCT03588078	Phase I/II	APR-246	2021
		TP53-mutant MDS	NCT03745716	Phase III	APR-246	2021
		TP53-mutant myeloid malignancies	NCT04214860	Phase I	Venetoclax and APR-246	2021
		AML, MDS	NCT02775903	Phase II	PD-L1 inhibitor durvalumab (MEDI4736)	2021
		AML, MDS	NCT03030612	Phase I/II	Anti-CD70 antibody ARGX-110	2021
		R/R AML, MDS	NCT01869114	Phase II	mTOR inhibitor sirolimus	2021
		Treatment-naïve MDS	NCT02942290	Phase I	Venetoclax	2022
		AML, MDS, CMML	NCT02397720	Phase II	PD-1 inhibitor nivolumab	2022
		AML, MDS	NCT04275518	Phase I	MDM2 inhibitor APG-115	2022
		AML, MDS	NCT02319369	Phase I	MDM2 inhibitor milademetan	2022
		AML, MDS, CMML	NCT04256317	Phase II/III	Cytidine deaminase inhibitor ASTX727	2023
		AML, MDS	NCT04609826	Phase I	Dihydroorotate dehydrogenase inhibitor JNJ-74856665	2023
		AML, MDS	NCT03113643	Phase I	Venetoclax and SL-401	2024
		AML, MDS, CMML, MPN	NCT03862157	Phase I/II	Venetoclax and pevonedistat	2024
		R/R AML, MDS	NCT04487106	Phase II	Venetoclax and trametinib	2024
		R/R FLT3-mutant AML, R/R MDS, R/R CMML, R/R MPN	NCT04140487	Phase I/II	Venetoclax and gilteritinib	2024
		AML, MDS, CMML	NCT04730258	Phase I/II	PLK4 inhibitor CFI-400945	2024
		AML, MDS, MPN	NCT04771130	Phase I/II	BCL2 inhibitor BGB-11417	2024
		AML, MDS with impending relapse	NCT04712942	Phase II	NEDD8-inhibitor pevonedistat	2026
	CC-486	AML, MDS after allo-HSCT	NCT04173533	Phase III		2024
	Decitabine	AML, MDS	NCT03066648	Phase I	PD-1 inhibitor PDR001 and checkpoint inhibitor MBG453	2021
		Untreated AML or R/R AML	NCT02878785	Phase I/II	PARP inhibitor talazoparib	2022
		AML, MDS, CMML	NCT03306264	Phase III	Cytidine deaminase inhibitor ASTX727	2022
		AML, MDS, CMML	NCT04730258	Phase I/II	PLK4 inhibitor CFI-400945	2024
		R/R AML, R/R high-risk MDS	NCT03404193	Phase II	Venetoclax	2024
		R/R AML, MDS	NCT02190695	Phase II	Carboplatin, arsenic trioxide	2026
	Guadecitabine (SGI-110)	AML, MDS, CMML	NCT01261312	Phase I/II		2019
		AML, MDS	NCT03603964	Phase II		2021
		AML and MDS after allo-HSCT	NCT03454984	Phase II		2022
		AML, MDS, CMML	NCT02935361	Phase I/II	PD-L1 inhibitor atezolizumab	2021
	NTX-301 (DNMT1 inhibitor)	AML, MDS, CMML	NCT04167917	Phase I		2025
BET	Birabresib (OTX015, MK-8628)	AML, sAML, DLBCL	NCT02698189	Phase I		2021
	CPI0610	AML, MDS, MPN	NCT02158858	Phase I/II	JAK1/2 inhibitor ruxolitinib	2021
	ABBV-744	R/R AML	NCT03360006	Phase I		2022
	FT-1101	R/R AML, MDS, non-Hodgkin’s lymphoma	NCT02543879	Phase I	Azacitidine	2019
	PLX2853	R/R AML, MDS	NCT03787498	Phase I		2021
	PLX51107	AML, MDS	NCT04022785	Phase I		2022
HDAC	LBH589 (Panobinostat)	AML, MDS, CMML	NCT00946647	Phase Ib/IIb	Azacitidine	2019
		High-risk AML and MDS after allo-HSCT	NCT04326764	Phase III		2023
	Vorinostat	AML, MDS	NCT00948064	Phase II	Azacitidine	2017
		AML and MDS after allo-HSCT	NCT03843528	Phase I	Low-dose azacitidine	2021
	Belinostat	R/R AML, R/R MDS	NCT03772925	Phase I	NEDD8-inhibitor pevonedistat	2021
IDH1	Ivosidenib	IDH1-mutant AML and MDS	NCT03503409	Phase II		2025
		IDH1-mutant AML	NCT03173248	Phase III	Azacitidine	2022
	BAY1436032	IDH1-mutant AML	NCT03127735	Phase I		2019
	FT-2102	IDH1-mutant AML and MDS	NCT02719574	Phase I/II	Azacitidine or cytarabine	2020
	IDH305	IDH1-R132 mutant AML and MDS	NCT02381886	Phase I		2022
	LY3410738	IDH1- or IDH2-mutant AML, MDS, CMML, MPN	NCT04603001	Phase I		2023
IDH2	Enasidenib	IDH2-mutant AML and MDS	NCT03744390	Phase II		2023
		IDH2-mutant AML and MDS	NCT03383575	Phase II	Azacitidine	2023
		IDH2-mutant AML, MDS, CMML after allo-HSCT	NCT04522895	Phase II		2024
EZH2	Tazemetostat	R/R Non-Hodgkin’s lymphoma	NCT03009344	Phase I		2020
		B-cell lymphomas, advanced solid tumors, DLBCL	NCT01897571	Phase I/II		2021
DOT1L	Pinometostat	R/R AML or AML with MLL-rearrangement	NCT03701295	Phase I/II	Azacitidine	2021
		AML with MLL-rearrangement	NCT03724084	Phase I/II	Standard chemotherapy	2021
PRMT5	GSK3326595	AML, MDS	NCT03614728	Phase I	Azacitidine	2023
	JNJ-64619178	Advanced solid tumors, non-Hodgkin’s lymphoma, low-risk MDS	NCT03573310	Phase I		2022
	PF-06939999	Advanced or metastatic solid tumors	NCT03854227	Phase I		2024
LSD1	Tranylcypromine	AML, MDS	NCT02273102	Phase I	ATRA	2020
	GSK2879552	AML, MDS	NCT02177812	Phase I	ATRA	2017
	IMG-7289	AML, MDS	NCT02842827	Phase I	ATRA	2018
	INCB059872	Solid tumors and AML, MDS	NCT02712905	Phase I/II	ATRA, azacitidinecitidine and nivolumab	2020
	Seclidemstat (SP-2577)	CMML, MDS	NCT04734990	Phase I/II	Azacitidine	2022
	CC-90011	R/R AML, treatment-naïve AML not eligible for chemotherapy	NCT04748848	Phase I/II	Venetoclax and azacitidine	2025

## Data Availability

Data sharing not applicable.
